# 二甲双胍抗肺癌机制研究进展

**DOI:** 10.3779/j.issn.1009-3419.2020.102.02

**Published:** 2020-04-20

**Authors:** 高祥 王, 美青 徐, 明然 解

**Affiliations:** 1 230001 合肥，安徽医科大学附属省立医院胸外科 Department of Thoracic Surgery, Anhui Provincial Hospital Affiliated to Anhui Medical University, Hefei 230001, China; 2 230001 合肥，中国科学技术大学附属第一医院胸外科 Department of Thoracic Surgery, The First Affiliated Hospital of USTC, Division of Life Sciences and Medicine, University of Science and Technology of China, Hefei 230001, China

**Keywords:** 肺肿瘤, 二甲双胍, 糖尿病, Lung neoplasms, Metformin, Diabetes mellitus

## Abstract

二甲双胍作为治疗2型糖尿病的一线用药，安全性及有效性得到证实。近年来流行病学研究发现二甲双胍具有抑制肺癌细胞增殖及转移等特性，有望成为一种新的抗肺癌药物。肺癌是一种严重危害人类健康的疾病，其发病率和死亡率一直居所有恶性肿瘤之首，且预后差。近年来大量证据表明二甲双胍能降低肺癌等肿瘤的发病风险及死亡率，其机制主要包括激活单磷酸腺苷活化的蛋白激酶通路、改善高胰岛素血症及胰岛素抵抗、促进肺癌细胞凋亡、抑制相关炎症反应等。本文就二甲双胍对肺癌的研究做一综述。

## Abstract (en)

Metformin, as a first-line drug in the treatment of type 2 diabetes, has been proved to be safe and effective. In recent years, epidemiological studies have found that metformin can inhibit the proliferation and metastasis of lung cancer cells, and is expected to become a new anti-lung cancer drug. Lung cancer is a disease that seriously endangers human health, its morbidity and mortality have been ranked first among all malignant tumors, and the prognosis is poor. In recent years, a great deal of evidence shows that metformin can reduce the risk and mortality of tumors such as lung cancer. Its mechanisms mainly include activating adenosine monophosphate-activated protein kinase pathway, improving hyperinsulinemia and insulin resistance, promoting lung cancer cell apoptosis and inhibiting related inflammatory response. The aim of this article is to reviews the study of metformin on lung cancer.

肺癌发病率和死亡率在全世界范围内一直居于所有恶性肿瘤之首^[1]^，而非小细胞肺癌（non-small cell lung cancer, NSCLC）是肺癌中最常见的一种类型，约占肺癌的80%-85%^[2]^。而随着全世界人口老龄化的进程，肺癌合并糖尿病的患者数量呈逐年上升的趋势。糖尿病是最常见内分泌疾病之一，而糖尿病中2型糖尿病占据90%以上^[3, 4]^。近年相关研究^[5-7]^发现，2型糖尿病与胃癌、结直肠癌、乳腺癌等多种恶性肿瘤的发生发展相关。另外，国内外相关研究报道，2型糖尿病会增加肺癌患者的发病风险及死亡风险^[8, 9]^。2型糖尿病影响肺癌患者预后的机制可能与高糖血症、高胰岛素血症及胰岛素抵抗等有关，而二甲双胍作为治疗2型糖尿病的一线用药，其具有价格低廉且药毒性低等优点。近年来，大量流行病学及临床研究揭示二甲双胍具有抗肿瘤特性，现将二甲双胍抗肺癌的相关作用及机制作一综述。

## 1 二甲双胍与肺癌关系的临床研究

二甲双胍作为治疗2型糖尿病的一线降糖药之一，文献报道其能够降低肺癌患者的死亡率，增加化疗药物的效果及改善肺癌患者的生存情况^[10]^。Lin等^[11]^通过比较服用二甲双胍与未服用二甲双胍的Ⅳ期NSCLC合并糖尿病患者的总生存率发现，二甲双胍组的中位生存期为5个月，而未用二甲双胍治疗的患者为3个月。倾向得分匹配分析表明，在控制了社会人口统计、糖尿病严重程度、其他糖尿病药物、癌症特征和治疗后，二甲双胍组的存活率显著改善。Wink等^[12]^通过对682例同步放化疗的糖尿病合并晚期非小细胞肺癌患者分析发现，单独使用二甲双胍患者无进展生存期和无远处转移生存期明显优于未使用二甲双胍患者，且二甲双胍的使用是同步放化疗晚期非小细胞肺癌患者的独立预后因素。Xu等^[13]^通过回顾性研究发现，接受二甲双胍（1, 000 mg, *bid*）治疗的患者的中位总生存期和无病生存时间比没有接受二甲双胍治疗的患者的中位总生存期和无病生存时间明显延长。多因素分析显示二甲双胍治疗可独立预测糖尿病NSCLC的远期预后。Arrieta等^[14]^通过进行一项前瞻性2期临床试验发现，总共139例Ⅲb期-Ⅳ期肺腺癌患者随机分配接受网络表皮生长因子受体酪氨酸激酶抑制剂（epidermal growth factor receptor-tyrosine kinase inhibitors, EGFR-TKIs）或EGFR-TKIs加二甲双胍（500 mg, *bid*）治疗，EGFR-TKIs+二甲双胍组的中位无进展生存期明显长于EGFR-TKIs组，接受联合治疗的患者的中位生存期也明显延长。

## 2 二甲双胍抗肺癌可能相关机制

### 2.1 激活AMPK信号通路

腺苷酸活化蛋白激酶（AMP-activated protein kinase, AMPK）由一个催化α亚基和两个调节亚基β和γ组成^[15]^，并且是细胞对低能量反应的保守调节因子，当细胞内出现氧化应激、缺氧和低血糖等细胞刺激时，细胞内三磷酸腺苷（adenosine triphosphate, ATP）浓度降低而一磷酸腺苷（adenosine monophosphate, AMP）浓度升高时，AMPK通路被激活^[16]^。肝激酶B1（liver kinase B1, LKB1）本质为一种丝氨酸/苏氨酸蛋白酶，且是AMPK的关键调节因子，LKB1/AMPK信号通路在细胞凋亡中起关键作用^[17]^。已有研究^[18]^表明，二甲双胍抑制线粒体呼吸链复合物Ⅰ，导致低细胞能量状态从而激活LKB1/AMPK信号通路，使抑癌基因*p53*磷酸化，增加蛋白质激酶p21的表达，从而达到抑制肺癌细胞的增殖及转移。AMPK还通过调节其下游信号通路雷帕霉素靶蛋白（mammalian target of rapamycin, MTOR）活性来控制癌细胞的生长、增殖和自噬，而这种活性在癌细胞中一直被解除调控^[19]^。Luo等^[20]^通过实验发现，二甲双胍（8 mmol/L）主要通过AMPK/PKA/GSK-3β信号通路抑制Survivin蛋白酶的表达，二甲双胍抑制蛋白激酶A（protein kinase, PKA）的活性，并诱导其下游糖原合成酶激酶3β（glycogen synthase kinase 3β, GSK-3β）的激活从而使Survivin蛋白水平下调。Survivin作为凋亡抑制蛋白的一员，在细胞死亡的调控中发挥着重要作用，其水平下降会诱导人肺癌细胞系的凋亡细胞毒性，从而使肺癌细胞凋亡^[20]^。

### 2.2 改善高胰岛素血症及胰岛素抵抗

糖尿病影响肺癌患者生存的一个主要机制为高胰岛素血症及胰岛素抵抗，而二甲双胍作为一种胰岛素增敏剂通过降低肺癌患者体内的血糖及胰岛素水平和改善患者体内的胰岛素抵抗从而达到抗肺癌的作用。胰岛素样生长因子-1受体（insulin-like growth factors-1 receptor, IGF-1R）是促进肿瘤发展的重要因子，研究^[21, 22]^表明二甲双胍能够抑制IGF-1R信号传导而达到抗肿瘤的作用。Li等^[23]^研究发现二甲双胍能够通过降低IGF-1R的传导活化能力而增强了H2228和H3122细胞对克唑替尼的敏感性，此外，在对克唑替尼耐药的H2228-CR和H3122-CR细胞中，二甲双胍的应用逆转了其耐药性。

### 2.3 促进肺癌细胞凋亡

二甲双胍通过激活JNK/p38MAPK通路和GADD153抑制肺癌细胞的生长并诱导凋亡^[24]^。MAPK是一种丝氨酸/苏氨酸蛋白酶，其两个亚族JNK及p38MAPK在细胞凋亡中起关键作用。二甲双胍通过激活JNK/p38MAPK信号通路，从而达到抑制肺癌细胞生长并诱导其凋亡^[24]^。另外，*GADD153*是一种凋亡基因，长时间的使用二甲双胍刺激肺癌细胞株，肺癌细胞内该基因的表达会上调，从而增加肺癌细胞发生凋亡的机会^[24]^。Wu等^[24]^研究发现，人肺腺癌A549细胞和NCI-H1299细胞在二甲双胍（4 mmol/L及8 mmol/L）的干预下，其生长增殖受到抑制，8 mmol/L抑制细胞增殖效果更强，二甲双胍能够促进A549细胞凋亡。另外，将A549细胞移植到裸鼠身上，注射二甲双胍依然可抑制肿瘤细胞增殖，并且未观察到任何毒副作用或小鼠体质量改变。最近Luo等^[20]^研究发现二甲双胍（8 mmol/L）通过AMOK/PKa/GSK-3β轴介导的c-FLIP降解而杀死非小细胞肺癌细胞。c-FLIP是细胞凋亡途径的关键负性调节因子。c-FLIP在各种肿瘤和细胞系中总是过度表达，包括肺癌，过度表达的c-FLIP增加了对TRAIL和Fas介导的癌细胞凋亡的抵抗力，而二甲双胍通过降低c-FLIPL蛋白的稳定性而显著抑制c-FLIPL的表达，从而增加癌细胞的凋亡^[25]^。

### 2.4 抑制相关炎症反应

慢性炎症与肿瘤的发生及发展密切相关，其中包括炎症细胞及炎症因子。抑制癌症患者体内的炎症反应可以破坏癌症细胞的有利环境。有文献^[26]^报道，这些炎症介质能够相互作用，从而增加癌细胞的发生及发展。晚期糖基化终产物（advanced glycation end products, AGE）与晚期糖基化终产物受体（receptor for advanced glycation end products, RAGE）结合在糖尿病并发症、肺癌发病和转移的发病机制中起主导作用，而RAGE激活诱导细胞因子[肿瘤坏死因子α（tumor necrosis factor α, TNF-α）、白介素-1（interleukin-1, IL-1）、白介素-6（interleukin-6, IL-6）、转化生长因子（transforming growth factor, TGF）]分泌触发炎症途径，从而导致肺癌细胞的发生、增殖及转移等^[27]^。二甲双胍能够抑制炎症因子的表达和破坏癌细胞的微环境，从而抑制癌细胞的生长及转移。

## 3 二甲双胍与肺癌关系的体内和体外研究

目前对二甲双胍抗肿瘤机制仍没有明确，大部分的体外实验及动物实验揭示，二甲双胍可以抑制肿瘤细胞的生长及促进肿瘤细胞凋亡，从侧面说明二甲双胍本身存在着直接的抗肿瘤作用，而不是通过降低患者体内血糖水平间接达到的抗肿瘤作用。Wu等^[24]^体内研究试图通过临床应用剂量的二甲双胍应用于荷瘤小鼠，实验结果表明：二甲双胍在较低剂量（40 mg/kg/d）时对体内移植瘤的生长有20%的抑制作用，但不如较高剂量（200 mg/kg/d, 41%）那么强。二甲双胍治疗过程中未观察到对荷瘤小鼠的明显毒性作用，这表明二甲双胍具有相对有利的毒性^[24]^。Kurimoto等^[28]^研究发现用TGF-β和成纤维细胞生长因子-2（fibroblast growth factor-2, FGF-2）诱导PC-9和HCC-827腺癌细胞分化，PD-L1水平升高并增加肺腺癌细胞对吉非替尼和顺铂的耐药性，然而二甲双胍处理的PC-9和HCC-827腺癌细胞抑制PD-L1的表达并且能够逆转对吉非替尼和顺铂的耐药性。Li等^[29]^研究发现，用二甲双胍处理的PC-9和PC-9GR细胞会使其肺纤维化标志物减少，并伴有TGF-β激活减少和下游信号分子COL1A1、pSMAD2、pSMAD3、pSTAT3、pAKT和pERK降低。另外Li等^[29]^还发现，用300 mg/kg二甲双胍治疗Sprague Dawley大鼠可减轻吉非替尼诱导的肺纤维化恶化，肺纤维化会增加癌症风险。Groenendijk等^[30]^发现，注射了A549细胞的Balb/c小鼠用400 mg/kg/d的二甲双胍和30 mg/kg/d的索拉非尼治疗40 d，结果显示癌细胞增殖减少，肿瘤缩小，AMPK磷酸化增加，mTOR信号传导抑制，反映了二甲双胍具有抗肿瘤的特性。

## 4 小结与展望

目前流行病学研究显示二甲双胍对2型糖尿病患者中肺癌的发生具有潜在的保护机制且能改善肺癌合并2型糖尿病患者的预后。但对于正常人及未合并2型糖尿病的肺癌患者的临床研究基本没有。另外，现有临床研究存在多种设计不足，如对二甲双胍使用剂量不一、未对研究人群进行更细化的分层等，从而影响了现有临床研究的可信度。分子生物学研究提示二甲双胍对肺癌细胞的影响具有多层次性。因此积极完善相关分子生物学研究是极为必要的。

综上所述，二甲双胍作为治疗2型糖尿病的一线药物，安全性及有效性已经得到证实。目前研究发现二甲双胍具有抗肺癌作用，在体外及体内实验也已证明二甲双胍能够抑制肺癌细胞的增殖，诱导肺癌细胞凋亡，但是其抗肺癌的机制存在多样性，还有待进一步的实验研究。另外，将二甲双胍作为抗肺癌的一线用药或辅助用药还存在很多问题需要解决及说明。
